# Supplementary material for: Neurons exploit stochastic growth to rapidly and economically build dense dendritic arbors
Source: Nat Commun. 2025 Jul 1;16:5903. doi: 10.1038/s41467-025-60800-7 (PMC12217211; doi:10.1038/s41467-025-60800-7)
Supplement: Supplementary file 1 — Supplementary Information [file 41467_2025_60800_MOESM1_ESM.pdf]

Supplementary Information

Neurons exploit stochastic growth to rapidly and economically build dense dendritic arbors

Xiaoyi Ouyang[1,2*], Sabyasachi Sutradhar[1*], Olivier Trottier[1,2,3], Sonal Shree[1], Qiwei Yu[4,5], Yuhai Tu[5], Jonathon Howard[1,2,6]✉

[1]Department of Molecular Biophysics and Biochemistry, Yale University, New Haven, CT 06511, USA.
[2]Department of Physics, Yale University, New Haven, CT 06511, USA.
[3]Current address: Department of Chemical and Physical Sciences, University of Toronto - Mississauga, Toronto, ON M5S 1A1, Canada
[4]Lewis-Sigler Institute for Integrative Genomics, Princeton University, Princeton, NJ 08544, USA.
[5]Center for Computational Biology, Flatiron Institute, New York, NY 10010, USA.
[6]Quantitative Biology Institute, Yale University, New Haven, CT 06511, USA.
[*]These authors contributed equally
✉Corresponding author. Email: joe.howard@yale.edu

Table of Contents

1. Pseudocode of directed-rod simulation procedure **2**

2. Full model including terminal-internal dendrite interconversion **3**

Supplementary Figures **7**

Supplementary Fig. 1: Extended data on branch length and radial angle distributions. 7

Supplementary Fig. 2: Terminal-internal branch interconversion following branching and debranching events. 8

Supplementary Fig. 3: Directed-rod simulations and determinations of collision pre-factors. 9

Supplementary Fig. 4: Relaxation towards the steady state. 10

Supplementary Fig. 5: early-stage asymmetric growth and late-stage tiling. 11

Supplementary Fig. 6: Supplementary on phase diagram. 12

Supplementary Fig. 7: Examples of directed-rod simulations over time. 13

Supplementary Fig. 8: Supplementary histograms on radial orientation. 14

Supplementary Fig. 9: Schematics of the full model with terminal-internal branch conversion. 15

1. Pseudocode of directed-rod simulation procedure

Inputs:
T_{sim}: Total simulation time in minutes
Δt : Small time step (~0.01 min)
L : Size of the square box (~200 μm)
N_{i} : Number of initial rods
k_{b} : branching rate constant (μm^{-1} min^{-1})
k_{ij} : Transition rate between states
$V_{\mathrm{G}}, V_{\mathrm{P}}, V_{\mathrm{S}}$: Mean velocities for Growing, Paused and Shrinking states

Initialization:

i. Create a square box of size L with periodic boundaries.
ii. Put random bases $(x_i^{\mathrm{b}}, y_i^{\mathrm{b}})$ within the box for the tips.
iii. Set the length of tips $l_{\mathrm{i}} = 0$ in a growing state.
iv. Set random orientations for the tips $\theta_i \in [0, 2\pi]$

Simulation loop:
For t=0 to T_{sim} with step Δt:

1. *Branching event:*
Branching events are treated separately because it is independent of the tip state (G, P, S).
For each tip:

i. Compute branching probability:
$$P_i^{\mathrm{b}} = 1 - e^{-l_i(t) k_{\mathrm{b}} \Delta t}$$
ii. Compare P_i^{b} with a uniform random number $R1 \in (0,1)$
iii. If $R1 < P_i^{\mathrm{b}}$, nucleate a new tip in growing state with a random orientation and random position within the box.

2. *Tip Transition:*

i. Compute the total transition rate:
$$k_{\mathrm{tot}} = \sum_{j=(\mathrm{G,P,S})}^{j \neq i} k_{ij}$$
ii. Compute transition probability:
$$P_i = 1 - e^{-k_{\mathrm{tot}} \Delta t}$$
iii. Compare P_i with a uniform random number $R2 \in (0,1)$
iv. If $R2 < P_i^{\mathrm{b}}$, transition the tip state comparing another uniform random number $R3$ with each k_{ij}/k_{tot} to execute the proper state and assign corresponding velocity V_{G}, V_{P} or V_{S}.

3. *Growth/shrinkage*:

i. Update tip lengths:
$$l_i(t) = l_i(t - \Delta t) + V_{(\mathrm{G,P,S})} \Delta t$$
ii. Update tip endpoints:
$$x_i^{\mathrm{t}}(t) = x_i^{\mathrm{b}} + l_i(t)\cos(\theta_i)$$
$$y_i^{\mathrm{t}}(t) = y_i^{\mathrm{b}} + l_i(t)\sin(\theta_i)$$

4. *Collision check:*
Detect intersection between tips and remove the tip that intersects other tips.
Stopping condition:
If $t < T_{\mathrm{sim}}$ repeat steps 1 to 4, otherwise terminate the simulation.

2. Full model including terminal-internal dendrite interconversion

Here, we present a full model including internal dendrite density as a fourth species. As described in the main text, terminal and internal branches interconvert following branching and debranching (Supplementary Fig. 2). Branching from a terminal branch will "sever" it into two shorter branches: the proximal part becomes a terminal branch, and rest becomes an internal branch (Supplementary Fig. 2a). Conversely, debranching, either through collision or spontaneously, fuses a pair of adjacent terminal and internal branches to form a longer terminal branch (Supplementary Fig. 2b-c). If branching or debranching occurs alongside an internal branch, it only breaks or joins internal branches. While this process converts internal branch density within its own kind, it does not involve interconversion with terminal branches.

For proof of principle, we first present a minimal model that includes terminal-internal interconversion based on the one-state model. This eliminates spontaneous disappearance by assuming constant growth for all terminal branches. Without loss of generality, we further assume that branching and debranching occur exclusively on terminal branches. Ignoring the transport term, the equations can be written as follows:

$$\frac{\partial n_{\mathrm{T}}(l,t)}{\partial t} = -\bar{v}\frac{\partial n_{\mathrm{T}}(l,t)}{\partial l} - K_{\mathrm{col}}(t)n_{\mathrm{T}}(l,t) + \mathcal{T}_{\mathrm{I}\to\mathrm{T}}(n_{\mathrm{T}}, n_{\mathrm{I}}, l) \tag{1}$$

$$\frac{\partial n_{\mathrm{I}}(l,t)}{\partial t} = \mathcal{T}_{\mathrm{T}\to\mathrm{I}}(n_{\mathrm{T}}, n_{\mathrm{I}}, l) \tag{2}$$

The dendrite densities of the terminal and internal branches are denoted by n_{T} and n_{I}, respectively. $\bar{v}$ represents the drift velocity in the one-state model. The remaining terms are $\mathcal{T}_{\mathrm{I}\to\mathrm{T}}(n_{\mathrm{T}}, n_{\mathrm{I}}, l)$ and $\mathcal{T}_{\mathrm{T}\to\mathrm{I}}(n_{\mathrm{T}}, n_{\mathrm{I}}, l)$, which represent terminal-internal branch conversions. More specifically, $\mathcal{T}_{\mathrm{I}\to\mathrm{T}}(n_{\mathrm{T}}, n_{\mathrm{I}}, l)$ represents net conversion from terminal branches of all lengths to internal branch of specific length l. Similarly, $\mathcal{T}_{\mathrm{T}\to\mathrm{I}}(n_{\mathrm{T}}, n_{\mathrm{I}}, l)$ represents net conversion from internal branches of all lengths to terminal branch of specific length l.

Terminal branches with length l are severed at the same rate of branching from dendrites with length l, which takes place with rate $k_{\mathrm{b}}l$. Assuming random branching locations, the probability density of having a severed branch of length $l' < l$ is given by $k_{\mathrm{b}}l\, n_{\mathrm{T}}(l) \times \frac{1}{l} = k_{\mathrm{b}}n_{\mathrm{T}}(l)$. Summing over all possible $l > l'$, we find $k_{\mathrm{b}} \int_{l}^{\infty} n_{\mathrm{T}}(l')dl'$ to be the total production rate of terminal or internal branch with length l' severed from a longer terminal branch. Meanwhile, after each collision event, a terminal branch and an internal branch are randomly selected, weighted by their respective dendrite densities, to be joined. For a combined length of l, the rate of terminal branches formed following debranching is proportional to the convolution of $n_{\mathrm{T}}(l)$ and $n_{\mathrm{I}}(l)$, i.e. $\int_0^l n_{\mathrm{T}}(l')n_{\mathrm{I}}(l-l')dl'$. With proper normalizations, the conversion terms $\mathcal{T}_{\mathrm{I}\to\mathrm{T}}(n_{\mathrm{T}}, n_{\mathrm{I}}, l)$ and $\mathcal{T}_{\mathrm{T}\to\mathrm{I}}(n_{\mathrm{T}}, n_{\mathrm{I}}, l)$ satisfy:

$$\mathcal{T}_{\mathrm{I}\to\mathrm{T}}(n_{\mathrm{T}}, n_{\mathrm{I}}, l) = -k_{\mathrm{b}}l\, n_{\mathrm{T}} + k_{\mathrm{b}}\int_{l}^{\infty} n_{\mathrm{T}}(l')dl' - K_{\mathrm{col}}(t)n_{\mathrm{T}} + K_{\mathrm{col}}(t)\frac{\int_0^l n_{\mathrm{T}}(l')n_{\mathrm{I}}(l-l')dl'}{N_{\mathrm{I}}} \tag{3}$$

$$\mathcal{J}_{\mathrm{T}\to\mathrm{I}}(n_{\mathrm{T}}, n_{\mathrm{I}}, l) = k_{\mathrm{b}} \int_{l}^{\infty} n_{\mathrm{T}}(l')dl' - K_{\mathrm{col}}(t)\frac{N_{\mathrm{T}}}{N_{\mathrm{I}}} n_{\mathrm{I}} \tag{4}$$

Given Supplementary Eq. (3) and (4), Supplementary Eq. (1)-(2) are solved at the steady state using the Laplace transform to convert the length variable l to the Laplace variable s.

$$\bar{l}_{\mathrm{T}} - \frac{\bar{v}}{k_{\mathrm{b}}} s g_{\mathrm{T}} - \bar{l}_{\mathrm{T}} g_{\mathrm{T}} + \left[\frac{dg_{\mathrm{T}}}{ds} + \left(\frac{1}{s} - \frac{g_{\mathrm{T}}}{s}\right) - \bar{l}_{\mathrm{T}} g_{\mathrm{T}} + \bar{l}_{\mathrm{T}} g_{\mathrm{T}} g_{\mathrm{I}}\right] = 0 \tag{5}$$

$$\frac{1}{s} - \frac{g_{\mathrm{T}}}{s} - \bar{l}_{\mathrm{T}} g_{\mathrm{I}} = 0 \tag{6}$$

Here, $g_{\mathrm{T}}(s) \equiv \mathcal{L}[n_{\mathrm{T}}(l)]/N_{\mathrm{T}}$ and $g_{\mathrm{I}}(s) \equiv \mathcal{L}[n_{\mathrm{I}}(l)]/N_{\mathrm{I}}$ denote the Laplace transform of n_{T} and n_{I} at steady state, normalized by N_{T} and N_{I}, respectively. When $s = 0$, notice that $\mathcal{L}[n_{\mathrm{T}}(l)]_{s=0} = \int_0^\infty n_{\mathrm{T}}(l)\, dl \equiv N_{\mathrm{T}}$ and $\mathcal{L}[n_{\mathrm{I}}(l)]_{s=0} = \int_0^\infty n_{\mathrm{I}}(l)\, dl \equiv N_{\mathrm{I}}$, hence $g_{\mathrm{T}}(s=0) = g_{\mathrm{I}}(s=0) = 1$. Assuming terminal and internal number densities are initially identical, we can set $N_{\mathrm{T}} = N_{\mathrm{I}}$ at all times since they vary with the same rate. More specifically, $dN_{\mathrm{T}}/dt = dN_{\mathrm{I}}/dt = k_{\mathrm{b}}\rho_{\mathrm{T}} - K_{\mathrm{col}}(t)N_{\mathrm{T}}$. After eliminating g_{I} in Supplementary Eq. (5) with Supplementary Eq. (6), we substitute g_{T} with an exponential ansatz in Laplace space $g_{\mathrm{T}}(s) = A/(A+s)$. As a result, it must satisfy that:

$$\left(\bar{l}_{\mathrm{T}} - \frac{\bar{v}A}{k_{\mathrm{b}}}\right) + \left(1 - 2\bar{l}_{\mathrm{T}}A + \frac{\bar{v}}{k_{\mathrm{b}}}A^2\right)\frac{1}{A+s} = 0 \tag{7}$$

For Supplementary Eq. (7) to be valid for all s, expressions within both brackets must vanish. This requires that $\bar{l}_{\mathrm{T}} = \sqrt{\bar{v}/k_{\mathrm{b}}}$ and $A = 1/\bar{l}_{\mathrm{T}} = \sqrt{k_{\mathrm{b}}/\bar{v}}$, hence $g_{\mathrm{T,SS}}(s) = 1/(1 + \bar{l}_{\mathrm{T}}s)$ at steady state (SS). Substituting $g_{\mathrm{T}}(s)$ with its steady-state expression in Supplementary Eq. (6) reveals that $g_{\mathrm{I}}(s)$ has the same expression as $g_{\mathrm{T}}(s)$. Taking the inverse Laplace transform immediately yields that terminal and internal branches share an identical exponential distribution:

$$n_{\mathrm{T,SS}}(l) = n_{\mathrm{I,SS}}(l) = \frac{N_{\mathrm{T}}}{\bar{l}_{\mathrm{T}}} \exp\left(-\frac{l}{\bar{l}_{\mathrm{T}}}\right) \tag{8}$$

Plugging Supplementary Eq. (8) into Eq. (3) and (4), it becomes apparent that $\mathcal{J}_{\mathrm{I}\to\mathrm{T}}(n_{\mathrm{T}}, n_{\mathrm{I}}, l) = \mathcal{J}_{\mathrm{T}\to\mathrm{I}}(n_{\mathrm{T}}, n_{\mathrm{I}}, l) = 0$. This result signifies that the terminal-internal interconversions reach equilibrium for any given length l at steady state.

To investigate the stability of this exponential solution, we added a small perturbation term around the steady state, i.e. $n_{\mathrm{T}}(l,t) = n_{\mathrm{T,SS}}(l) + m_{\mathrm{T}}(l,t)$ and $n_{\mathrm{I}}(l,t) = n_{\mathrm{I,SS}}(l) + m_{\mathrm{I}}(l,t)$, where $m_{\mathrm{T}}(l,t) \ll n_{\mathrm{T,SS}}(l)$ and $m_{\mathrm{I}}(l,t) \ll n_{\mathrm{I,SS}}(l)$. To keep the total branch number unchanged, we required $\int_0^\infty m_{\mathrm{T}}(l,t)\, dl = \int_0^\infty m_{\mathrm{I}}(l,t)\, dl = 0$. With proper linearization, we obtained the differential equations of the perturbation terms $m_{\mathrm{T}}(l,t)$ and $m_{\mathrm{I}}(l,t)$.

$$\frac{1}{k_{\mathrm{b}}}\frac{\partial m_{\mathrm{T}}}{\partial t} = -\bar{l}_{\mathrm{T}}^2 \frac{\partial m_{\mathrm{T}}}{\partial l} - 2\bar{l}_{\mathrm{T}} m_{\mathrm{T}} - l m_{\mathrm{T}} + \int_l^\infty m_{\mathrm{T}}(l')dl' + \int_0^l [m_{\mathrm{T}}(l') + m_{\mathrm{I}}(l')] e^{-\frac{l-l'}{\bar{l}_{\mathrm{T}}}} dl' \tag{9}$$

$$\frac{1}{k_\mathrm{b}}\frac{\partial m_\mathrm{I}}{\partial t} = \int_l^\infty m_\mathrm{T}(l')dl' - \bar{l}_\mathrm{T} m_\mathrm{I} \tag{10}$$

Taking the Laplace transform of Supplementary Eq. (9) and (10) results in the following matrix-form equation:

$$\frac{1}{k_\mathrm{b}}\frac{\partial}{\partial t}\begin{pmatrix}\widetilde{m}_\mathrm{T}\\ \widetilde{m}_\mathrm{I}\end{pmatrix} = \underbrace{\begin{pmatrix}-2\bar{l}_\mathrm{T} - \bar{l}_\mathrm{T}^2 s - \dfrac{1}{s(\bar{l}_\mathrm{T}s+1)} & \dfrac{\bar{l}_\mathrm{T}}{\bar{l}_\mathrm{T}s+1}\\ -\dfrac{1}{s} & -\bar{l}_\mathrm{T}\end{pmatrix}}_{M}\begin{pmatrix}\widetilde{m}_\mathrm{T}\\ \widetilde{m}_\mathrm{I}\end{pmatrix} + \begin{pmatrix}-\dfrac{\partial \widetilde{m}_\mathrm{T}}{\partial s}\\ 0\end{pmatrix} \tag{11}$$

$\widetilde{m}_\mathrm{T}(s,t)$ and $\widetilde{m}_\mathrm{I}(s,t)$ are the Laplace transform of $m_\mathrm{T}(l,t)$ and $m_\mathrm{I}(l,t)$. For $s > 0$, it is straightforward to identify that $\mathrm{tr}M = -\bar{l}_\mathrm{T}\left[3 + \bar{l}_\mathrm{T}s + 1/[\bar{l}_\mathrm{T}s(\bar{l}_\mathrm{T}s+1)]\right] < 0$ and $\det M = \bar{l}_\mathrm{T}^2\left[2 + \bar{l}_\mathrm{T}s + 2/\left[\bar{l}_\mathrm{T}s(\bar{l}_\mathrm{T}s+1)\right]\right] > 0$. It indicates $\widetilde{m}_\mathrm{T}(s) = \widetilde{m}_\mathrm{I}(s) = 0$ as a global stable fixed point for a dynamical system governed by the matrix M with any $s > 0$. The additional differential term $-\partial\widetilde{m}_\mathrm{T}/\partial s$ only shifts $\widetilde{m}_\mathrm{T}$ positively along the s axis, while having no effect on the total amount of $\widetilde{m}_\mathrm{T}$. Consequently, both $\widetilde{m}_\mathrm{T}(s)$ and $\widetilde{m}_\mathrm{I}(s)$ converge to zero over time. In other words, $m_\mathrm{T}(l,t)$ and $m_\mathrm{I}(l,t)$ must converge to zero for all l as $t \to \infty$. Hence the exponential steady-state solution is globally stable. To verify this stability, numerical solutions of Supplementary Eq. (1) and (2) are calculated with the forward Euler method from different initial conditions, up to 2000 minutes with a time step of 0.01 minutes (Supplementary Fig. 9a). Both terminal and internal dendrite densities converge to the predicted exponential distributions as shown in Supplementary Eq. (8). Figure insets in Supplementary Fig. 9a demonstrate the convergence of terminal-internal interconversion to zero for all branch length l, as previously predicted.

To examine these conclusions in a full model based on the three-state model, we first extend Main Text Eq. (1)-(3) to incorporate a fourth species: internal dendrite density.

$$\frac{\partial n_\mathrm{G}(r,l,\theta,t)}{\partial t} = -(k_\mathrm{GS}+k_\mathrm{GP})n_\mathrm{G} + k_\mathrm{SG}n_\mathrm{S} + k_\mathrm{PG}n_\mathrm{P} - v_\mathrm{G}\frac{\partial n_\mathrm{G}}{\partial l} - K_\mathrm{col}(r,t)n_\mathrm{G} - \frac{v_\mathrm{G}}{2}\mathcal{R}(r,\theta)n_\mathrm{G} + \mathcal{T}_{\mathrm{I}\to\mathrm{G}}(n_\mathrm{G},n_\mathrm{I},l) \tag{12}$$

$$\frac{\partial n_\mathrm{S}(r,l,\theta,t)}{\partial t} = k_\mathrm{GS}n_\mathrm{G} - (k_\mathrm{SG}+k_\mathrm{SP})n_\mathrm{S} + k_\mathrm{PS}n_\mathrm{P} + v_\mathrm{S}\frac{\partial n_\mathrm{S}}{\partial l} + \frac{v_\mathrm{S}}{2}\mathcal{R}(r,\theta)n_\mathrm{S} + \mathcal{T}_{\mathrm{I}\to\mathrm{S}}(n_\mathrm{S},n_\mathrm{I},l) \tag{13}$$

$$\frac{\partial n_\mathrm{P}(r,l,\theta,t)}{\partial t} = k_\mathrm{GP}n_\mathrm{G} + k_\mathrm{SP}n_\mathrm{S} - (k_\mathrm{PG}+k_\mathrm{PS})n_\mathrm{P} + \mathcal{T}_{\mathrm{I}\to\mathrm{P}}(n_\mathrm{P},n_\mathrm{I},l) \tag{14}$$

$$\frac{\partial n_\mathrm{I}(r,l,\theta,t)}{\partial t} = \mathcal{T}_{\mathrm{G}\to\mathrm{I}}(n_\mathrm{G},n_\mathrm{I},l) + \mathcal{T}_{\mathrm{S}\to\mathrm{I}}(n_\mathrm{S},n_\mathrm{I},l) + \mathcal{T}_{\mathrm{P}\to\mathrm{I}}(n_\mathrm{P},n_\mathrm{I},l) + \mathcal{T}_{\mathrm{I}\leftrightarrow\mathrm{I}}(n_\mathrm{I},l) \tag{15}$$

This full model is illustrated in Supplementary Fig. 9b. Following the same arguments as previously discussed in the minimal full model, we can explicitly write out the interconversion terms in Supplementary Eq. (12)-(15) as:

$$\mathcal{T}_{\mathrm{I}\to\mathrm{X}}(n_\mathrm{X}, n_\mathrm{I}) = -k_\mathrm{b} l\, n_\mathrm{X} + k_\mathrm{b}\int_l^\infty n_\mathrm{X}(l')dl' - K_\mathrm{disap}(t) n_\mathrm{X} + K_\mathrm{disap}(t)\frac{\int_0^l n_\mathrm{X}(l') n_\mathrm{I}(l-l')dl'}{N_\mathrm{I}} \quad (16)$$

$$\mathcal{T}_{\mathrm{X}\to\mathrm{I}}(n_\mathrm{X}, n_\mathrm{I}) = k_\mathrm{b}\int_l^\infty n_\mathrm{X}(l')dl' - K_\mathrm{disap}(t)\frac{N_\mathrm{X}}{N_\mathrm{I}} n_\mathrm{I} \quad (17)$$

$$\mathcal{T}_{\mathrm{I}\leftrightarrow\mathrm{I}}(n_\mathrm{I}) = -k_\mathrm{b} l\, n_\mathrm{I} + 2k_\mathrm{b}\int_l^\infty n_\mathrm{I}(l')dl' - 2K_\mathrm{disap}(t) n_\mathrm{I} + K_\mathrm{disap}(t)\frac{\int_0^l n_\mathrm{I}(l') n_\mathrm{I}(l-l')dl'}{N_\mathrm{I}} \quad (18)$$

Here, $\mathrm{X} \in (\mathrm{G}, \mathrm{S}, \mathrm{P})$ and $K_\mathrm{disap}(t) \equiv [K_\mathrm{col}(t) N_\mathrm{G} + (1-\beta) n_\mathrm{S}(l=0) v_\mathrm{S}]/N_\mathrm{tot}$ is the net rate of branch disappearance, combining collision-based and spontaneous catastrophe. Using the steady-state condition that branching equals debranching, $k_\mathrm{b}\rho_\mathrm{tot} = K_\mathrm{disap} N_\mathrm{tot}$, one can verify that the exponential solution $n_\mathrm{X,SS} = N_\mathrm{X}/\bar{l}\exp(-l/\bar{l})$ and $n_\mathrm{I,SS} = N_\mathrm{T}/\bar{l}\exp(-l/\bar{l})$ satisfy $\mathcal{T}_{\mathrm{I}\to\mathrm{X}}(n_\mathrm{X,SS}, n_\mathrm{I,SS}) = \mathcal{T}_{\mathrm{X}\to\mathrm{I}}(n_\mathrm{X,SS}, n_\mathrm{I,SS}) = \mathcal{T}_{\mathrm{I}\leftrightarrow\mathrm{I}}(n_\mathrm{I,SS}) = 0$. Again, this solution indicates that internal branches have the same length and exponential distribution as terminal branches. Thus, we can disregard the internal variable and terminal-internal interconversion terms, while setting $\rho_\mathrm{tot} = 2\rho_\mathrm{T}$ and $N_\mathrm{tot} = 2N_\mathrm{T}$. Supplementary Eq. (12)-(14) hereby reduce to Main Text Eq. (1)-(3) with the correction for internal branches.

Supplementary Figures

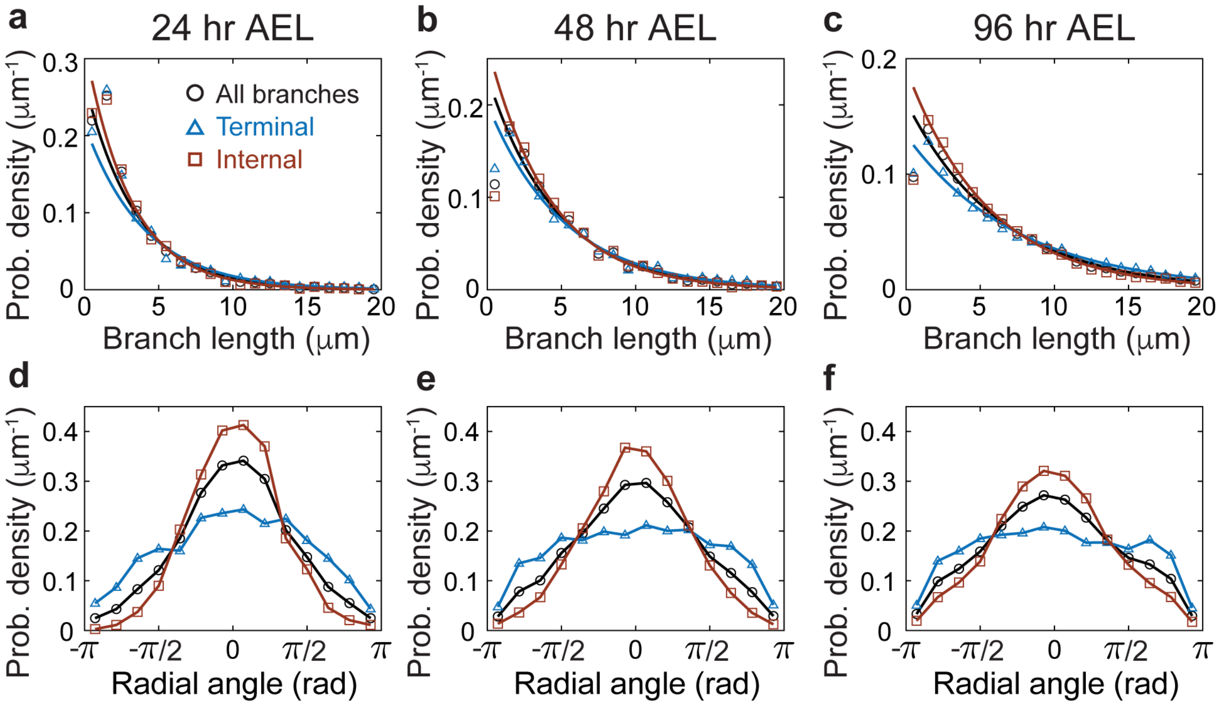

Supplementary Fig. 1: Extended data on branch length and radial angle distributions.
a-c Branch length distributions at 24, 48, and 96 hours AEL are shown with exponential fits (ignoring the first bin). **d-f** Radial angle distributions at 24, 48, and 96 hours AEL, respectively. Data from all branches are shown in black circles, from terminal branches are shown in blue triangles, and from internal branches are shown in brown rectangles.

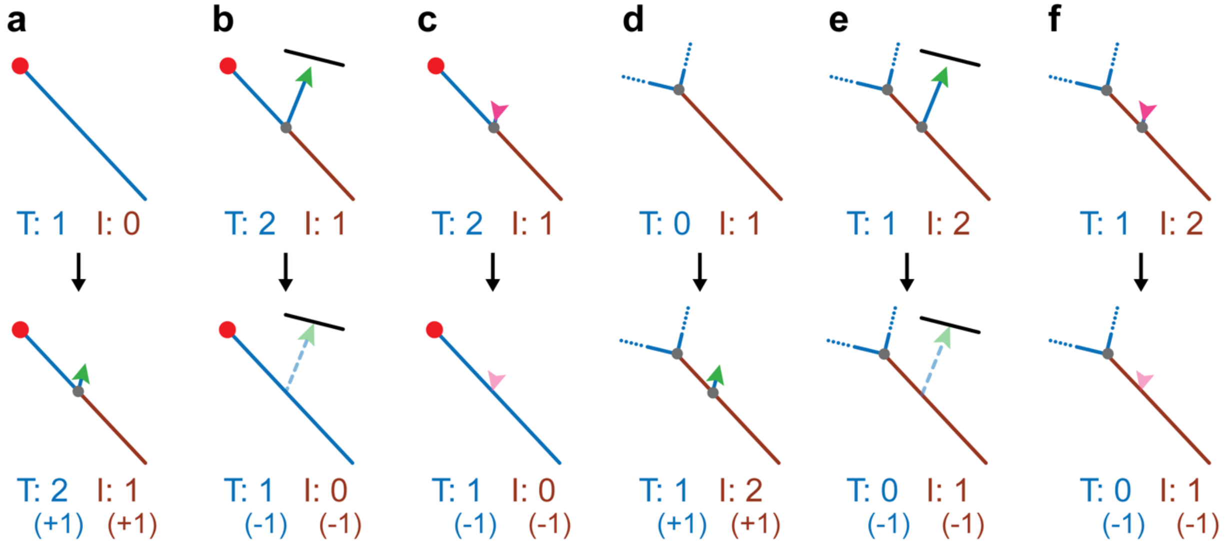

Supplementary Fig. 2: Terminal-internal branch interconversion following branching and debranching events.

a Branching alongside a terminal branch divides a terminal branch into a shorter terminal branch and an internal branch. **b**, **c** Debranching alongside a terminal branch, either from collision-based (**b**) or spontaneous (**c**) retraction, merges an internal and a terminal branch to form a longer terminal branch. **d-f** Branching or debranching alongside an internal branch only breaks or joins two internal branches. Terminal and internal branches do not interconvert in this case.

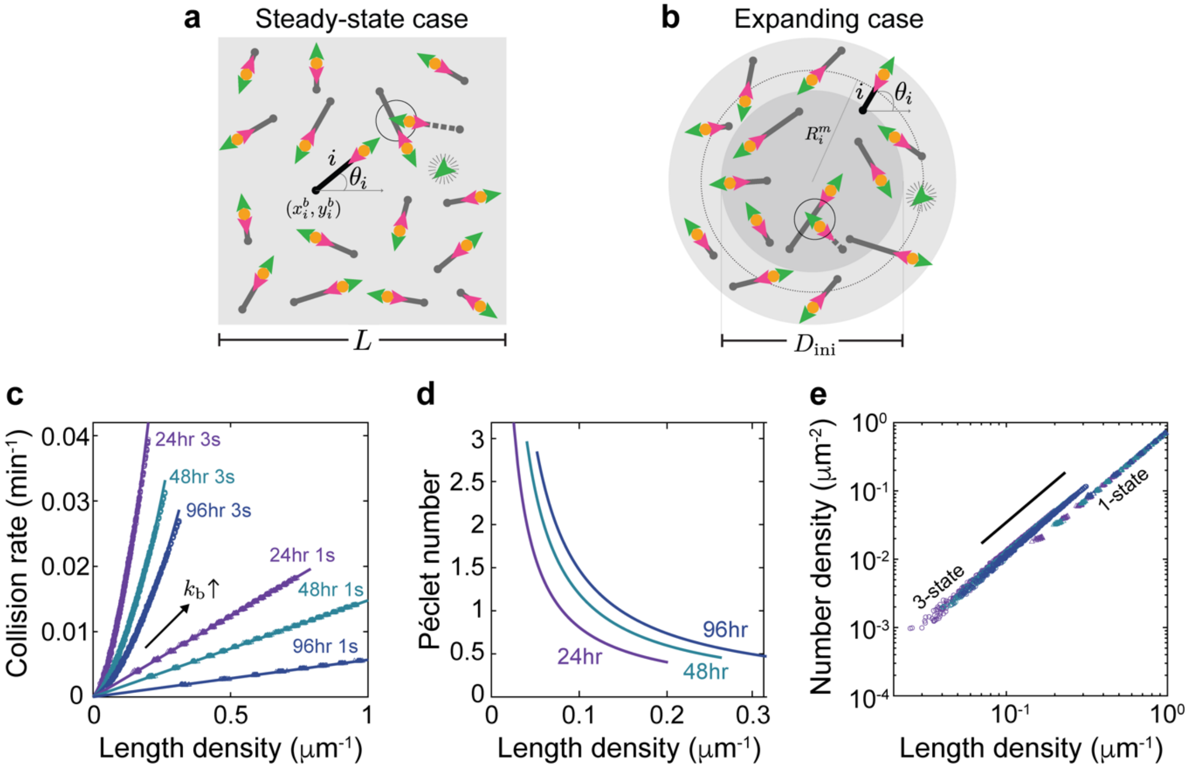

Supplementary Fig. 3: Directed-rod simulations and determinations of collision pre-factors.

a Simulation schematic for the steady state case. The green, orange, and magenta arrowheads represent the dynamics of the growing end arising from the transitions between growing, paused and shrinking states. The rods nucleate with a uniform distribution within the simulation box (gray square) and grow in random direction, as shown by the ith black tip. A rod is removed (dashed line) when its growing end collides with another existing rod (black circle). **b** Simulation schematic for the expanding case. The system is initialized by uniformly nucleating N_{ini} number of tips inside a circular region of diameter D_{ini}. Tip dynamics follows the same rules as in **a**, except branching. New branches (green arrowhead) are nucleated at a random location on the circle describing the middle point of the mother branch. **c** Collision rate as a function of length density for branching rates, k_b, ranging from 0.0005 to 0.01 using tip parameters at 24, 48, and 96 hours AEL (Table 1) for the three-state model (3s). The values of α and γ are 1.4-1.6 and 0.6 (Table 1). In contrast, for the one-state model (1s) where collision rates linearly correlate with length density, the geometric factor α is 0.75. **d** The Péclet number, defined by the ratio of advective to diffusive collisions, is plotted for the 3-state simulation in **c**. The Péclet number approaches 1 when branching rate k_b and rebranching probability β are near their experimentally measured values. This indicates that advection and diffusion contribute comparably to total collisions. **e** The parabolic relation between the number density and length density in one-state and 3-state simulations. The differences in the pre-factors suggest that one-state and 3-state branching networks have different structures.

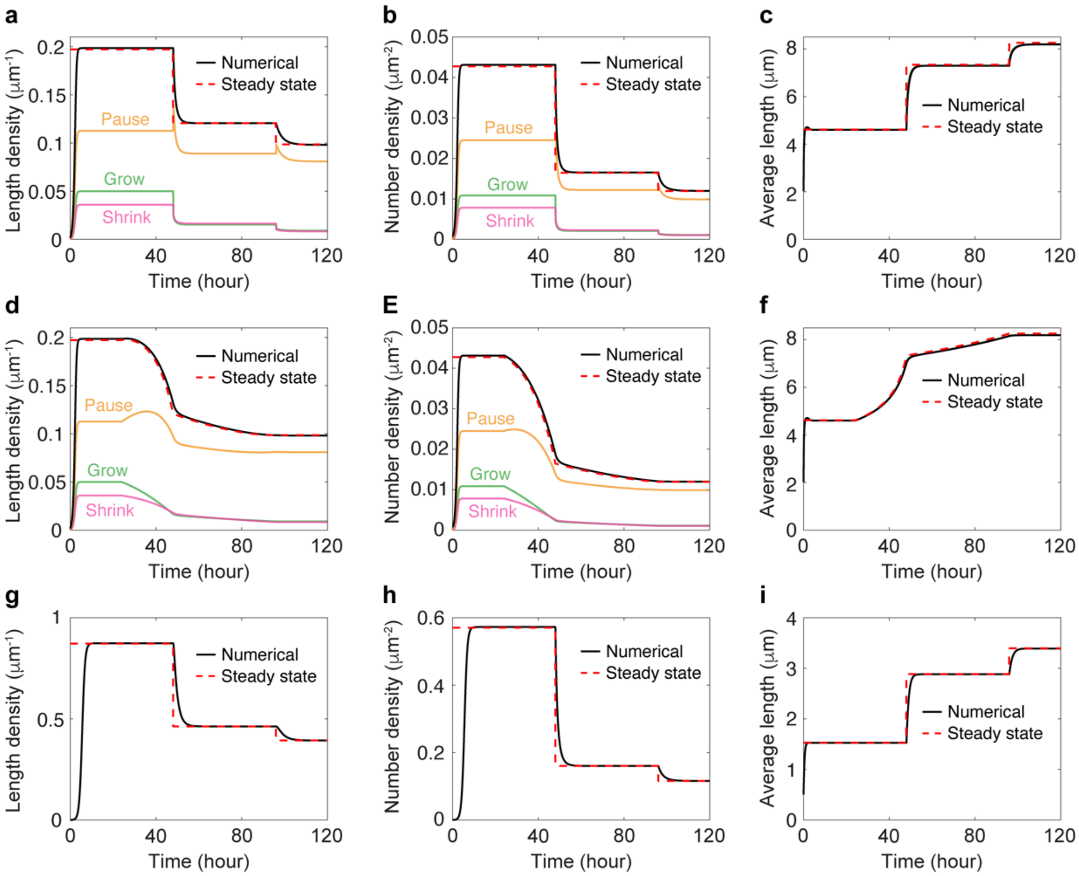

Supplementary Fig. 4: Relaxation towards the steady state.
a-c Numerical solutions of the length density, number density and average branch length in response to a sudden change in microscopic parameters introduced at 48 and 96 hours AEL. **d-f** Numerical solutions of the length density, number density and average branch length with a linear change in parameters from 24 to 48 and 48 to 96 hours AEL. **g-i** one-state numerical solutions of the length density, number density and average branch length with a sudden change in microscopic parameters introduced at 48 and 96 hours AEL.

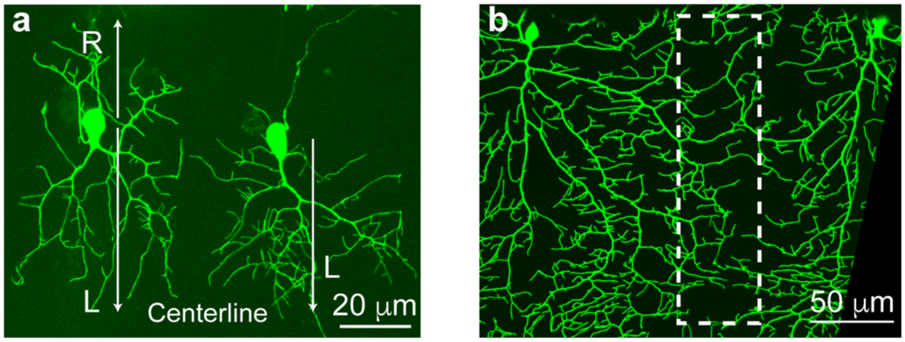

Supplementary Fig. 5: early-stage asymmetric growth and late-stage tiling.
a Examples of asymmetric growth of class IV neurons at 24 hours AEL. Both neurons exhibit faster growth along the left direction (towards the centerline for these examples). **b** Tiling of class IV neurons at 96 hours AEL.

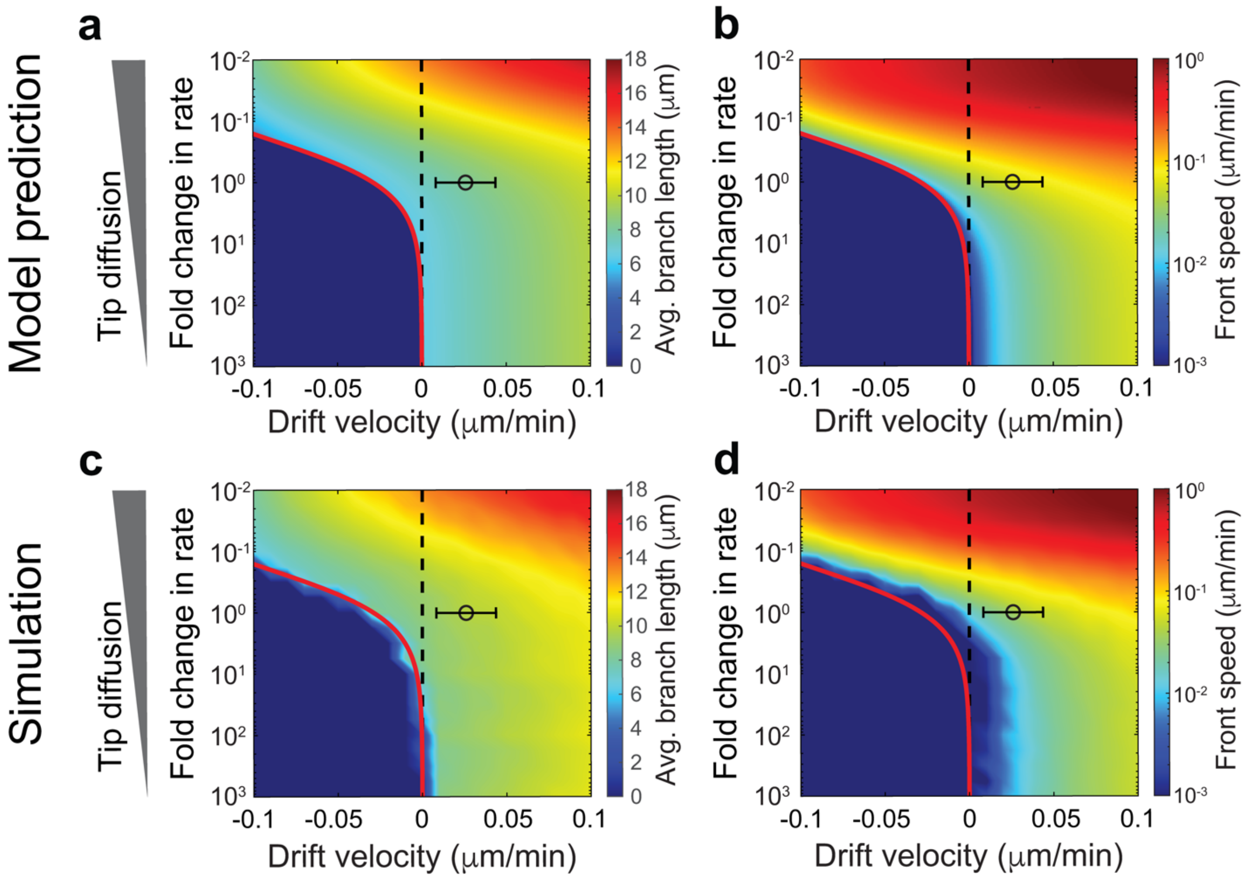

Supplementary Fig. 6: Supplementary on phase diagram.
a-b Phase diagrams of average branch length and front speed predicted by the model using 48-hour-AEL parameter set. The black circle represents experimental measurements for drift velocity and transition rates. Error bar is also included for drift velocity. **c-d** Phase diagrams of average branch length and front velocity from mean-field simulations within the same parameter space as **a** and **b**.

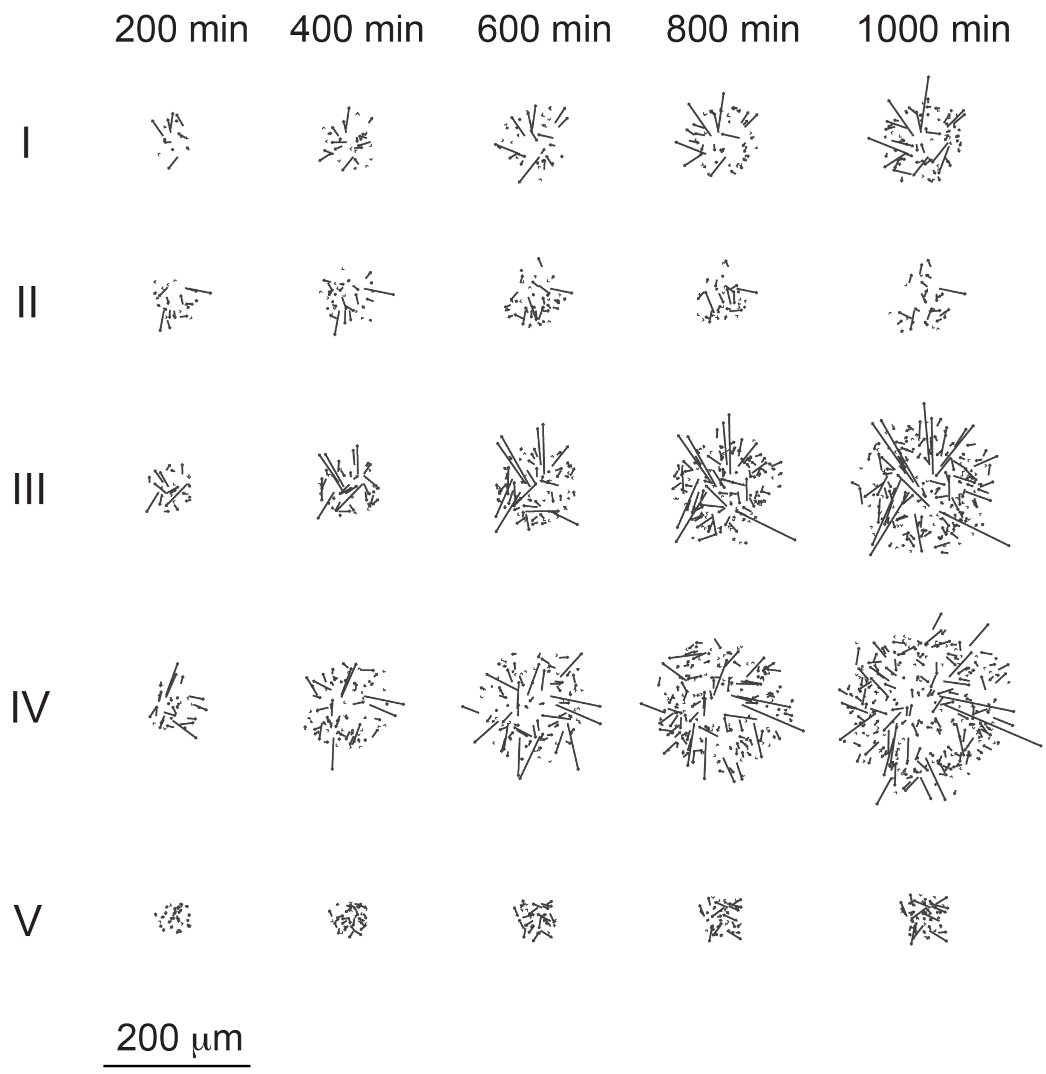

Supplementary Fig. 7: Examples of directed-rod simulations over time.
Examples of directed-rod configurations at 200, 400, 600, 800, and 1000 minutes using the parameters in Main Text Fig. 7b marked with I, II, III, IV, and V.

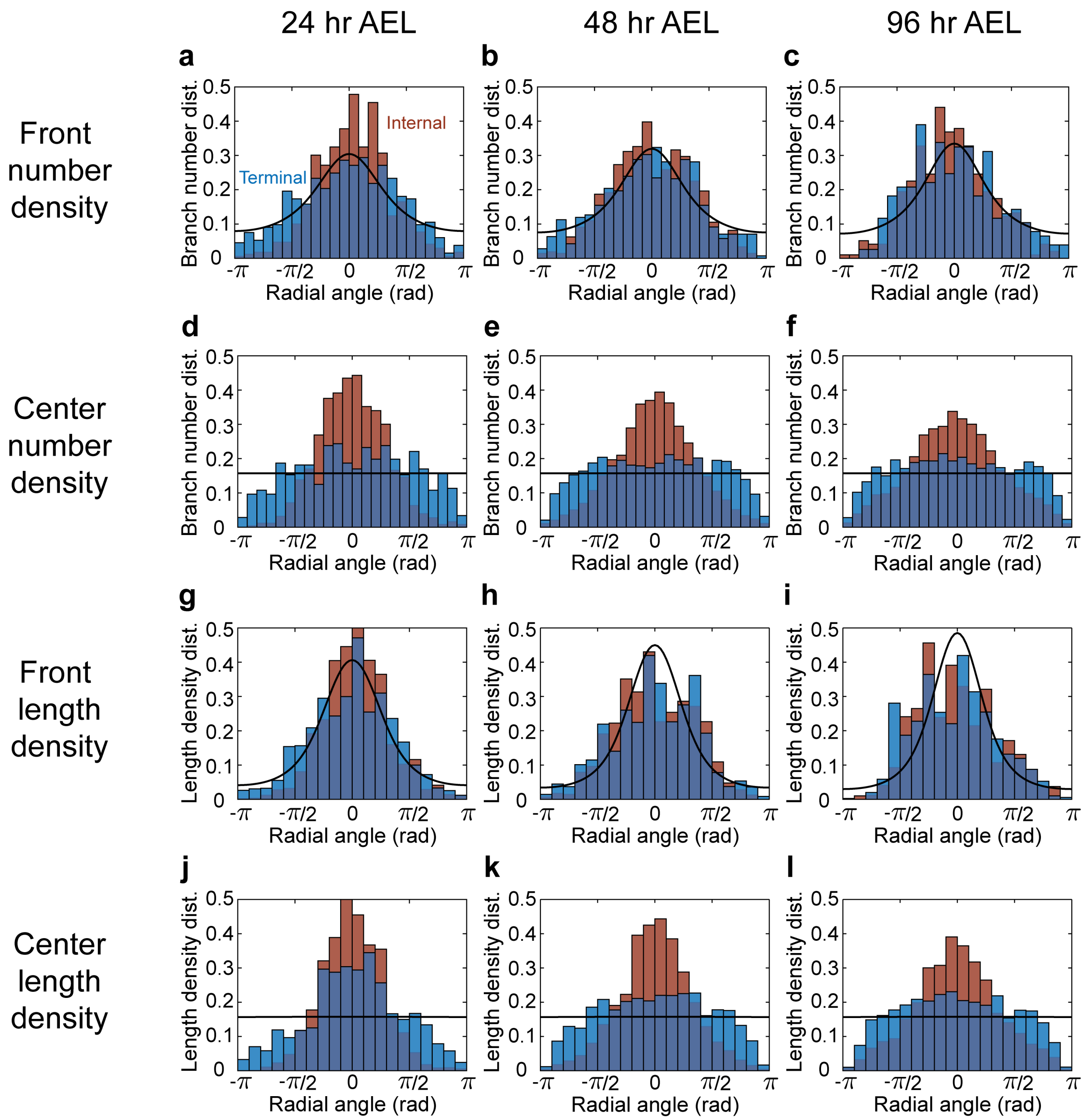

Supplementary Fig. 8: Supplementary histograms on radial orientation.
a-c Distributions of branch number density in the front at 24, 48, and 96 hours AEL. **d-f** Distributions of branch number density in the center at 24, 48, and 96 hours AEL. **g-i** Distributions of branch length density in the front at 24, 48, and 96 hours AEL. **j-l** Distributions of branch length density in the center at 24, 48, and 96 hours AEL. Throughout the figure, blue histograms show data from terminal branches, orange histograms show data from internal branches, and black curves represent model prediction from numerical solution.

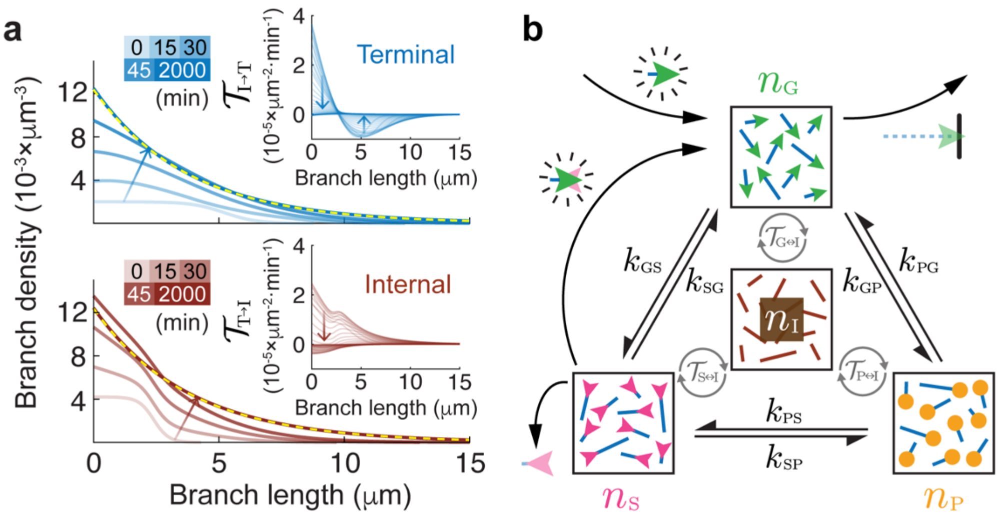

Supplementary Fig. 9: Schematics of the full model with terminal-internal branch conversion.

a Upper and lower panels showcase how terminal and internal dendrite densities evolve numerically from a sigmoidal initial distribution towards the predicted steady-state exponential distribution (yellow dashed curve). Density profiles are shown at 0, 15, 30, 45, and 2000 minutes with decreased transparency. Figure insets demonstrate convergence of interconversion terms $\mathcal{T}_{\mathrm{T}\to\mathrm{I}}$ and $\mathcal{T}_{\mathrm{I}\to\mathrm{T}}$ to zero for all branch lengths l with a time interval of 50 minutes. **b** Schematics of the mean-field model. Dendrite densities are illustrated by rods in boxes. Black bidirectional arrows demonstrate tip-state transitions between growth, shrinkage, and pause. Gray circular arrows signify branch interconversion. Contributions from branching, rebranching, collision-based, and spontaneous disappearance are shown with black arrows.
